# Data associated with a tellurium absorption atlas 19,000 – 24,000 cm^−1^

**DOI:** 10.1016/j.dib.2022.108038

**Published:** 2022-03-09

**Authors:** Amanda J. Ross, Joseph M. Cardon

**Affiliations:** a Institut Lumière Matière, Université Claude Bernard Lyon 1, CNRS, Université de Lyon, Villeurbanne F-69622, France; b Ideal Vacuum Products^1^, 5910 Midway Park Blvd NE, Albuquerque NM 87109, United States of America

**Keywords:** Te_2_ absorption, Tellurium atlas, Fourier transform spectrum of tellurium dimer, Te_2_ reference spectrum

## Abstract

Transmittance and absorbance data in the form of a spectral atlas have been obtained from Fourier transform spectra recorded on a commercial (Bomem DA3) instrument, operated with an external white light source injected through the instrument's `emission' port. The sample was a sealed, evacuated cell containing a small quantity of ^130^Te. This cell was placed in a ceramic furnace maintained close to 620 °C, with tellurium vapor pressures 8–11 Torr. The data are intended as an aid to spectral calibration in the blue-green to violet region. The data relate to work published in J. Mol. Spectrosc. 384 111,589 (2022).

**Specifications Table**

**Table:** 

Subject	Molecular Physics
Specific subject area	A spectral atlas; a reference spectrum for calibration extending from blue-green to violet.
Type of data	Figure (experimental setup) Figure (overview of raw spectra) Tables Processed wavenumber, transmittance and absorbance data. Raw absorption data (wavenumber, arbitrary intensity)
How the data were acquired	Spectra were recorded by Fourier transform interferometry [1], on a Bomem DA3.002 spectrometer operated with proprietary software (PCDA data acquisition). Light from an external quartz halogen white light source (Osram) running on a stabilized DC power supply (Fontaine) was collimated and passed through a 10 cm sealed evacuated cell, containing a small amount of ^130^Te (Ideal Vacuum Products). Interferograms were acquired at nominal apodized resolution of 0.02 to 0.033 cm^−1^. To achieve an acceptable signal-to-noise ratio, the spectrum was recorded in several pieces using combinations of optical filters, averaging between 200 (above 500 nm) and 600 scans (in the violet, where emission from the lamp was much weaker) in each piece.
Data format	Figure 1, setup: .pptx format Figure 2: spectrum: .jpg Processed: Ascii tables, 3 columns, .txt format Raw UV spectrum: ascii table, 2 columns, .txt format
Description of data collection	The tellurium cell was maintained at temperatures between 600 and 645 °C in a temperature-stabilised furnace [2] Δ*T* ± 7 °C, to provide strong absorption without saturation. External filters limited spectral extent, and a broadband glass filter (CS 4–97 or 5–61) in the FTS sample compartment removed residual HeNe contributions. Recording times were about 5 h per piece of spectrum*.*
Data source location	• **Institution:** Institut Lumière Matière, CNRS & Université Lyon 1 UMR 5306 • **City/Town/Region:** Villeurbanne • **Country:** France
Data accessibility	Available on the Mendeley Data repository, at https://data.mendeley.com/datasets/kmkbwtjhd3/1
Related research article	A.J. Ross and J. M. Cardon, Te_2_ absorption spectrum from 19,000 to 24,000 cm^−1^. J. Mol. Spectrosc. 384 (2022) 111,589 https://doi.org/10.1016/j.jms.2022.111589

## Value of the Data

• These data provide a convenient secondary spectral calibration standard for Doppler-limited applications in the blue/violet spectrum. They offer a convenient alternative to a printed atlas published in 1980 by Luc and Cariou [3].
• High-resolution spectroscopists working with tunable lasers can benefit from these data, particularly if working without a high-performance wavemeter.
• Part or all of the transmittance/absorbance files can be imported to any graphical software to enable direct comparison with calibration traces to establish an absolute wavenumber scale. The isotopically enriched tellurium source used here is commercially available. It is compact and convenient for secondary calibration.

## 1 Data description

Compressed Ascii data files and a graphical abstract are available on the Mendeley data base, at *this link (*https://data.mendeley.com/datasets/kmkbwtjhd3/1).

The file Te2_spectral_atlas.zip contains the atlas in three sections. The file Atlas_green.txt gives the transmittance (= transmission/baseline) and absorbance (= log_10_(1/transmittance)) recorded with external filters λ > 500 nm or bandpass centered at 506 nm, and internal filter Corning glass 4–97. It covers the range 18,850 to 20,400 cm^−1^.

The file Atlas_blue.txt gives the transmittance (= transmission/baseline) and absorbance (= log_10_ (1/transmittance)) recorded with external filters λ > 450 nm or bandpass centered at 460 nm, and internal filter Corning glass 5–61. It covers the range 20,100 to 22,450 cm^−1^.

The file Atlas_violet.txt gives the transmittance (= transmission/baseline) and absorbance (= log_10_ (1/transmittance)) recorded with external filter λ < 450 nm and internal filter Corning glass 5–61. It covers the range 22,400 to 24,015 cm^−1^.

The file Raw_transmission_spectrum_extending *to_*UV.zip is a transmission spectrum extending beyond the range of the 'official' atlas. It was recorded with the quartz uv beamsplitter. The intensity axis is arbitrary. Note that this spectrum extends beyond the 24,000 cm^−1^ limit indicated in the title. There is no check on calibration above 23,720 cm^−1^.

The file Te2_overviewFig.pdf provides a graphical abstract.

## 2 Experimental Design, Materials and Methods

**Fig. 1:**
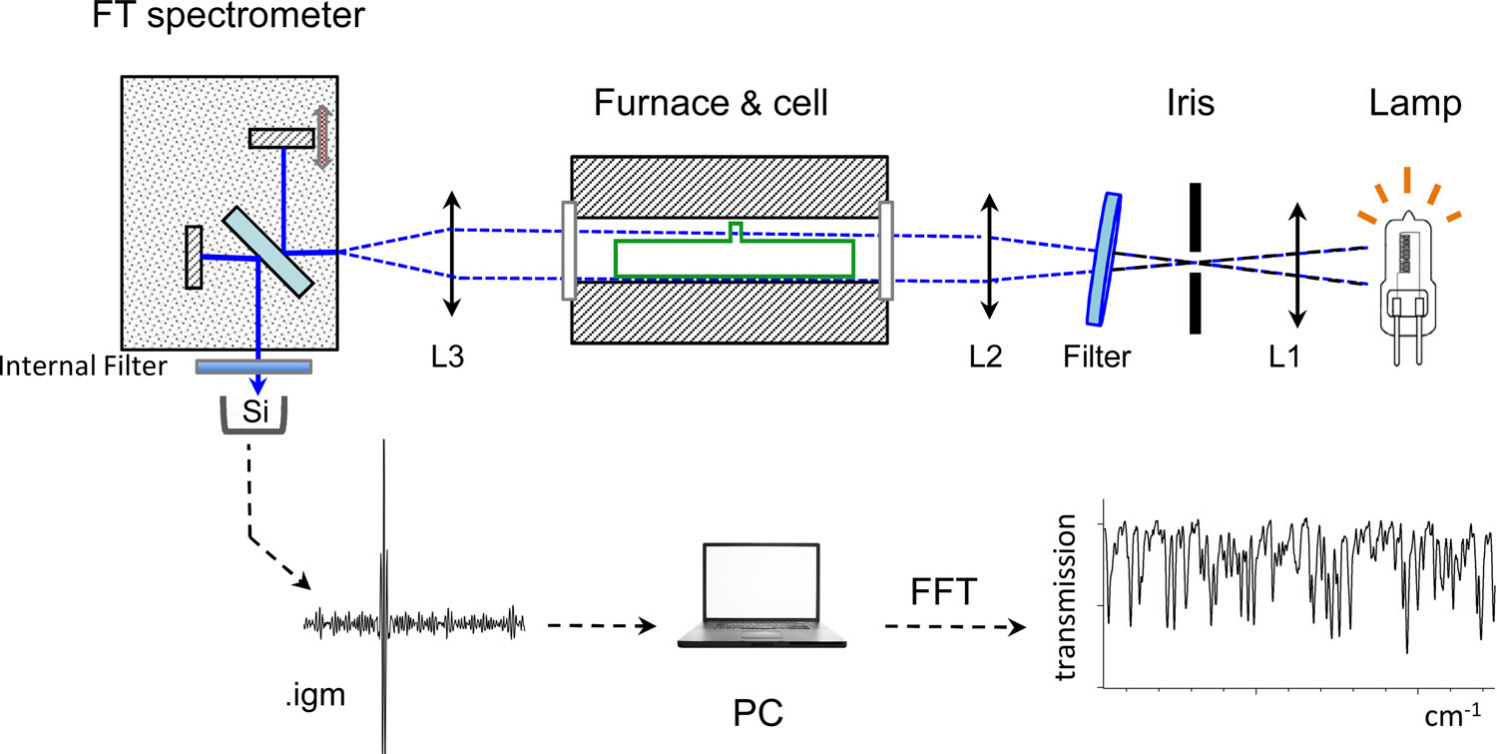
Diagram of the optical arrangement used to record the spectra. L1, L2 and L3 are convex lenses with focal lengths 4, 6 and 4 cm respectively, chosen to match the f/4 aperture of the FT interferometer and the physical diameter of the tellurium cell. The external filter is drawn tilted; we used combinations of interferential high- and low-pass filters from Corion Inc; their optical response can be (and was) tuned by changing the angle of incidence.

The experimental set-up used to record absorption spectra of tellurium dimer is illustrated schematically in Fig. 1. L1, L2 and L3 are convex lenses with focal lengths 4, 6 and 4 cm respectively, chosen to adapt to the f/4 aperture of the FT interferometer and to the physical diameter of the tellurium cell. The combination of the external and internal optical filters is intended to limit the spectral range to about 2000 cm^−1^, so that interferogram fringes (recorded on a silicon avalanche photodiode from Hamamatsu) were adapted to the dynamic range of the analog-to-digital converter, and so that the number of interferogram points remained below the PC-memory-imposed maximum of 10^6^. We aimed for 50% filling of the analog-to-digital converter at zero-path-difference by adjusting the current/voltage input to the external white light. The internal filter was a blue-green or blue band-pass filter, serving to remove stray HeNe light and attenuate the long-wavelength emission from the white-light source. We placed a Corning glass (cs 4–97, 5–56 or 5–61) filter inside the instrument sample compartment, in front of the detector. A variety of external filters were used. A series of narrow bandpass interference filters would have been ideal, but were available only centered at 520 and 460 nm. To cover the desired spectral region, we used combinations of an interferential low-pass filter, either λ < 450 or λ < 500 nm from Corion Inc. whose optical response is conveniently tuned by changing angle of incidence, and high-pass filters (Corning cs 3–73 or Corion λ > 450 nm). The tilt in Fig. 1 was actually close to 45° for the shortest wavelength region recorded with λ < 450 (Corion LS450) and Corning 5–61 filters).

**Fig. 2:**
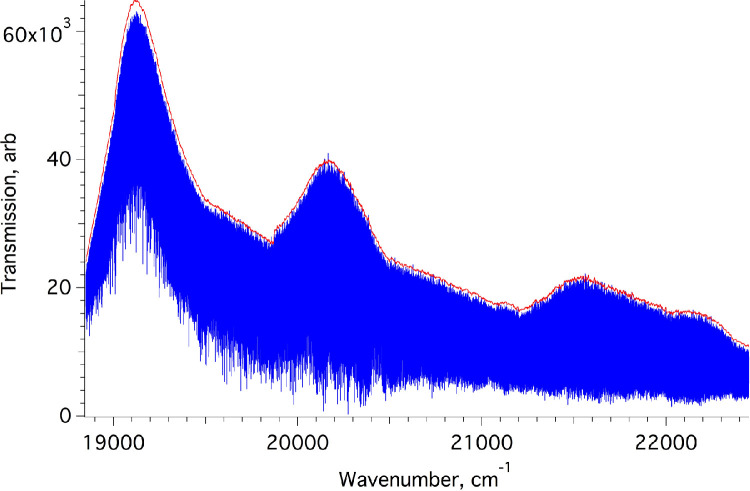
Overview of the composite transmission spectrum recorded with the visible beamsplitter, the contour being imposed by selections of optical filters. The continuous trace above (red) represents the baseline used to generate a transmittance spectrum.

The absorption spectra generated in Bomem GRAMS format were adjusted so that their wavenumber scale matched reference data from other sources [4], [5], [6]. A wavenumber-dependent correction was necessary, attaining 0.03 cm^−1^ at 23,000 cm^−1^. The pieces were then concatenated (see Fig. 2), averaging the regions of overlap using wave arithmetic software provided by IGOR-Pro 6 [7]. The percentile and boxcar smoothing algorithms in IGOR-Pro were used to generate the baseline contour required to generate transmittance and absorbance traces. Transmittance was calculated from Transmission/Baseline, and Absorbance as log_10_(1/Transmission). The results were exported using a cubic spline interpolation to generate Ascii files with evenly-spaced wavenumber points (a short extract is given in Table 1) covering the blue-green to violet region of the optical spectrum. The short wavelength end of the transmittance data is truncated at 24,015 cm^−1^, indicating the range for which we have confidence in the wavenumber scale. The fourth file available for download, Raw_transmission_spectrum_extending to_UV.zip contains only two columns, and provides the complete transmission curve for the last section of the spectrum, without truncation.

**Table 1:** Extract from the data file Atlas_green.txt, illustrating our data format. Column 1 is wavenumber in cm^−1^. Column 2 gives the transmittance (= transmission/baseline), and column 3 gives absorbance (=log_10_(1/transmittance)). The range and details of optical filters for each section of the atlas are given in the section `Data Description'.

Wavenumber, cm^−1^	Transmittance	Absorbance
18850.000	0.7677	0.1148
18850.002	0.7699	0.1136
18850.004	0.7713	0.1128
18850.006	0.7724	0.1122
18850.008	0.7739	0.1113
18850.010	0.7766	0.1098
18850.012	0.7812	0.1072
18850.014	0.7879	0.1035
18850.016	0.7962	0.0990
18850.018	0.8051	0.0941
18850.020	0.8136	0.0896
18850.022	0.8207	0.0858
18850.024	0.8257	0.0832
…..		

## Ethics Statements

The authors confirm that this paper is original work, adhering to the ethics principles of Data in Brief.

## CRediT authorship contribution statement

**Amanda J. Ross:** Investigation, Data curation, Writing – original draft, Writing – review & editing. **Joseph M. Cardon:** Methodology, Resources, Writing – review & editing.

## Declaration of Competing Interest

The authors declare the following financial interests/personal relationships which may be considered as potential competing interests. Although there is no obvious competing interest, we indicate that Joseph M. Cardon is an employee of Ideal Vacuum Products, a company that commercializes the tellurium reference cell and temperature-controlled furnace cited in the paper.
